# Identification of novel thiazole derivatives as flaviviral protease inhibitors effective against Dengue (DENV2) and Japanese encephalitis viruses

**DOI:** 10.1128/aac.01651-24

**Published:** 2025-02-24

**Authors:** Sheikh Murtuja, Sayan Das, Indrani Das Jana, Deepak Shilkar, Gourav Rakshit, Biswatrish Sarkar, Barij Nayan Sinha, Rikeshwer Prasad Dewangan, Arindam Mondal, Venkatesan Jayaprakash

**Affiliations:** 1 Department of Pharmaceutical Sciences & Technology, Birla Institute of Technology 309287 , Ranchi, Jharkhand, India; 2 Department of Bioscience and Biotechnology, Indian Institute of Technology 30112 , Kharagpur, West Bengal, India; 3 Department of Pharmaceutical Chemistry, School of Pharmaceutical Education and Research, Jamia Hamdard 428951 , New Delhi, India; IrsiCaixa Institut de Recerca de la Sida, Barcelona, Spain

**Keywords:** antivirals, NS2B-NS3 protease inhibitors, thiazoles, dengue virus, Japanese encephalitis virus

## Abstract

Flaviviruses are the causative agents of viral hemorrhagic fever (VHF) globally and have demonstrated the capacity to result in fatal outcomes if not managed effectively. Among different types of flaviviruses, dengue (DENV) and Japanese encephalitis (JEV) viruses are the most common in tropical and subtropical countries. While vaccines have been developed and licensed for both DENV and JEV, effective treatment options remain sparse. Hence, there is a pressing need to develop small molecules that can target machineries crucial for virus replication and remain conserved across different flaviviruses, thereby could serve as a promising therapeutic option. This study outlines the synthesis of novel thiazole compounds as flavivirus NS2B–NS3 protease inhibitor and characterization of their antiviral activity against DENV and JEV. We synthesized a heterocyclic template derived from a substrate-based retrotripeptide dengue protease inhibitor, leading to 48 thiazole derivatives. Two compounds, 3aq and 3au demonstrated significant inhibition of dengue virus protease activity *in vitro*. Comprehensive characterization of these two compounds was conducted through biochemical assay, which revealed an uncompetitive mode of inhibition. Subsequent cell-based assays using Dengue and Japanese encephalitis viruses as representative flaviviruses revealed the potential of these compounds to block viral RNA synthesis, and viral replication exhibiting 50% inhibitory concentrations (IC50s) in the low-micromolar range. Time-course experiments unveiled that the two compounds impeded the accumulation of viral genomic RNA primarily at later stages of infection, aligning with their capacity to hinder NS2B–NS3 protease activity, polyprotein processing and viral genomic RNA replication. Finally, time of addition experiment showed the compounds remain effective even when added 9 hpi, thereby confirming their potential as promising antivirals. Together, our work presents the development and validation of flavivirus protease inhibitors with therapeutic potential against Dengue (DENV2) and Japanese encephalitis viruses.

## INTRODUCTION

Flaviviruses belong to the Flaviviridae family and comprise nearly 70 pathogenic viruses, including Dengue virus (DENV), Japanese encephalitis virus (JEV), West Nile virus (WNV), Yellow fever virus (YFV), and Zika virus (ZIKV) (1, 2). These viruses are primarily arthropod-borne (arboviruses), transmitted through mosquitoes or ticks, and poses significant threat to global human health and economy (3, 4). The rising prevalence of DENV in tropical and subtropical regions, the occurrence of WNV in North America, and the spread of JEV across a substantial part of Asia and Oceania remain pressing issues (5). DENV is the most common arbovirus and is endemic to approximately 110 countries, with an estimated 400 million infections and 20,000 deaths annually (6). DENV causes dengue fever (DF), dengue hemorrhagic fever (DHF), and dengue shock syndrome (DSS) in humans (6, 7). The vaccines Dengvaxia (CYD-TDV, Sanofi Pasteur) and Qdenga (TAK-003, Takeda) have received regulatory approval; however, their effectiveness in controlling dengue epidemics has been limited (8). Rather, this vaccine elicits significant worries about antibody-dependent enhancement of illness (ADE) (9–11). Therefore, developing novel therapeutic approaches for DENV infections or creating broad-spectrum inhibitors for closely related flaviviruses is of utmost urgency (12).

DENV infection starts with receptor-mediated endocytosis of the virus particle, followed by the deposition of viral genetic material within the cytoplasm (6, 7). The genomic material of the virus is a 10.7-kb, capped, positive-sense RNA that is translated into a single polyprotein on the endoplasmic reticulum (ER) membrane. Both host and viral-encoded proteases subsequently process this polyprotein into three structural proteins, namely the capsid (C), membrane (M), and envelope (E), as well as seven non-structural proteins, namely NS1, NS2A, NS2B, NS3, NS4A, NS4B, and NS5 (13, 14). These newly synthesized viral proteins stimulate the transition of the viral genome to be used as a substrate for translation to a template for genomic RNA replication (15). Non-structural proteins, together with multiple host factors, constitute viral replication complexes that execute viral genomic RNA replication within infection-induced double-membrane vesicle packets (VP) generated from the ER membranes (14). The newly synthesized genomic RNA was packaged with different structural proteins along the host-derived membrane to generate premature and mature virion particles (14). The non-structural proteins NS2B and NS3 together constitute the viral serine protease, which is instrumental for polyprotein processing in the early phase of infection and virion maturation at later stages, thus acting as a critical modulator of the progression of infection (16). In the NS2B–NS3 complex, the NS3 protein constitutes the catalytic core, whereas NS2B acts as a cofactor important for stabilization and substrate recognition (17). The N-terminal domain of NS3 contains a highly conserved protease-active core consisting of His51, Asp75, and Serl35 triad motifs that are critical for enzymatic activity (18, 19). The NS2B–NS3 protease remains an attractive target for antiviral drug development due to its important role in flavivirus replication (20).

Viral proteases are well-accepted as effective targets for the discovery and development of antivirals (21). The clinical effectiveness of viral protease inhibitors has been established by incorporating these inhibitors as major components in antiretroviral therapy (22, 23). Although substrate-based peptidomimetics dominate protease inhibitor design, the low stability and bioavailability of peptide-based drugs have prompted the need for non-peptidic, small-molecule-based protease inhibitors (24, 25). Peptidic and non-peptidic inhibitors of DENV NS2B–NS3 reported were reviewed from time to time by us (26–28) and other scientific groups (29–32).

Here, we present the design, synthesis, and validation of two novel DENV protease inhibitors by transforming a retrotripeptide, previously reported by Weigel et al. (33). We synthesized 48 thiazole derivatives and conducted *in vitro* screening for their activity against DENV2 NS2B–NS3 protease. Two lead compounds, **3aq** and **3au**, featuring –OH and –OCH_3_ substitutions and displaying IC_50_ values below ~25 µM, were selected for further investigation regarding their antiviral efficacy against live DENV2 and JEV infections. Both compounds inhibited DENV2 and JEV viral growth significantly, but its efficacy against JEV is more drastic than DENV2 as demonstrated through IC_50_ and selectivity index (SI). Time-course experiments conclusively demonstrated that the compounds inhibited viral RNA replication solely at later stages of infection, proving their ability to inhibit protease activity, polyprotein processing, and the production of viral proteins. Finally, using time of addition experiment, we show that the compounds can effectively inhibit virus replication even when added 9 hour post-infection (hpi), thereby establishing the potential of these compounds as effective broad-spectrum therapeutic agents against multiple flaviviruses.

## RESULTS AND DISCUSSION

### Synthesis of thiazole derivatives

**Fig 1:**
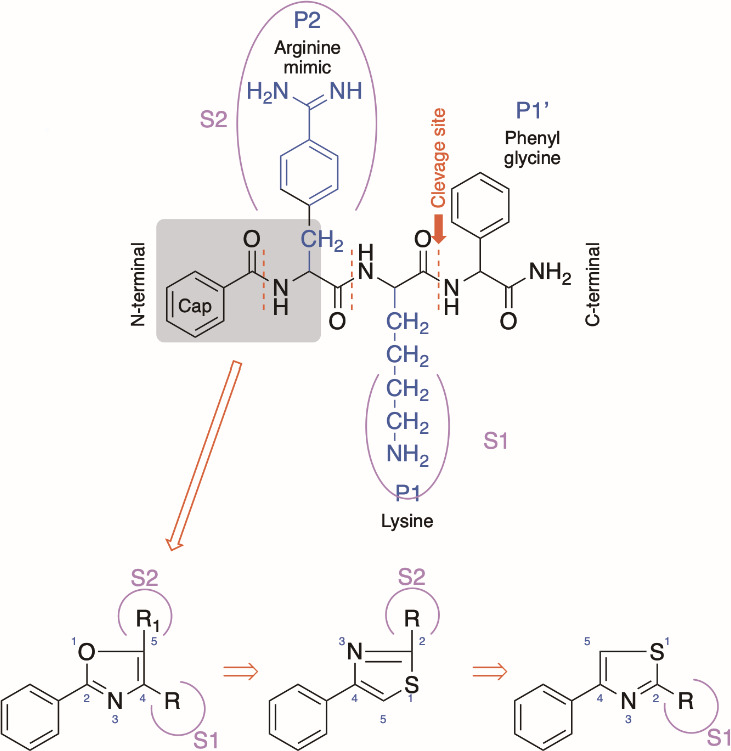
Strategy of designing small molecule inhibitors against dengue protease.

Flavivirus proteases prefer substrates containing dibasic amino acids (Lys or Arg) at the P1 and P2 positions on the non-prime side (34). Weigel et al. addressed this requirement by retaining lysine at the P1 position and replacing arginine at the P2 position with its mimic, *p*-acetamidinyl phenyl ring (33). In our design process, we evaluated the possibility of integrating an oxazole-based heterocyclic template on the N-terminal side of this retro-tripeptide (see Fig. 1). The design incorporates a 2-phenyl oxazole with substitutions at the 4th and 5th positions, strategically placed in the S1 and S2 subpockets of the enzyme, respectively. We replaced the oxazole ring with its structural counterpart, thiazole, to generate a thiazole derivative, which is well-documented for its antiviral properties (35, 36). Moreover, substitution at the second position of these thiazole molecules might occupy either the S1 or S2 subpockets due to rotational flexibility around the single bond linking the phenyl ring to the thiazole ring (Fig. 1).

**Fig 2:**
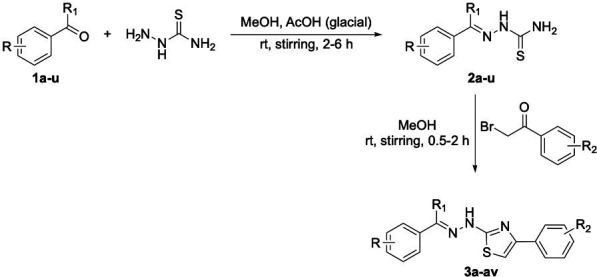
Synthesis of 2-[(2E)−2-benzylidene/-(1-phenylethylidene)-hydrazinyl]−4-phenyl-1,3-thiazole.

A set of 48 thiazole derivatives (**3a–3av**) was synthesized through the reactions illustrated in Fig. 2 as reported earlier (37). Suitably substituted benzaldehydes and acetophenones (**1a–1u**) were subjected to reaction with thiosemicarbazide in the presence of a catalytic quantity of glacial acetic acid in methanol. The reaction was conducted at room temperature with continuous stirring for about 4–6 h. The resulting thiosemicarbazones (**2a–2u**) were subsequently treated with appropriately substituted phenacyl bromide in methanol. The reaction was performed at room temperature under constant stirring for approximately 0.5–2 h.

The resultant thiazole derivatives (**3a–3av**) were purified through recrystallization from hot methanol. All final compounds were characterized by ^1^H-NMR, ^13^C-NMR, and electron spray ionization mass spectroscopy (ESI-MS). The protons of the methyl group (-CH_3_) at positions R (**3a**, **3b**, **3j**, **3s**, and **3t**) and R_1_ (**3s–3av**) appeared as singlets (s) with chemical shifts in the range of δ2.18–2.65 ppm. Additionally, a singlet (s) was observed in the range of δ3.74–3.95 ppm for the protons of the methoxy (-OCH_3_) group at R (**3c–3e, 3L–3n, 3w–3ab,** and **3ah–3ak**) and R_2_ (**3aq** and **3au**). The aromatic protons, including those in the thiazole ring (Ar-H), displayed multiplets in the range of δ6.20–9.91 ppm. Moreover, the vinylic proton at the R_1_ position (**3a–3r**) was detected in the aromatic region, coalescing with the aromatic proton peaks. A chemical shift ranging from δ9.03–9.87 ppm was observed for the proton of the -OH groups at R (**3u, 3v, 3af, 3ag,** and **3ao–3av**), appearing as a singlet. Additionally, for all compounds (**3a–3av**), the side chain -NH proton prominently appeared as a singlet within the range of δ10.96–12.56 ppm. ^13^C-NMR of compounds displayed peaks in the range of δ14.4–55.6 and δ99.2–171.0 ppm for aliphatic and aromatic carbons, respectively. All the compounds exhibited either [M]^+^ or [M + 1]^+^ as the base peak in ESI-MS. For halogenated compounds, [M + 2]^+^ peaks were also detected.

### *In vitro* screening for anti-flaviviral protease activity

**Fig 3:**
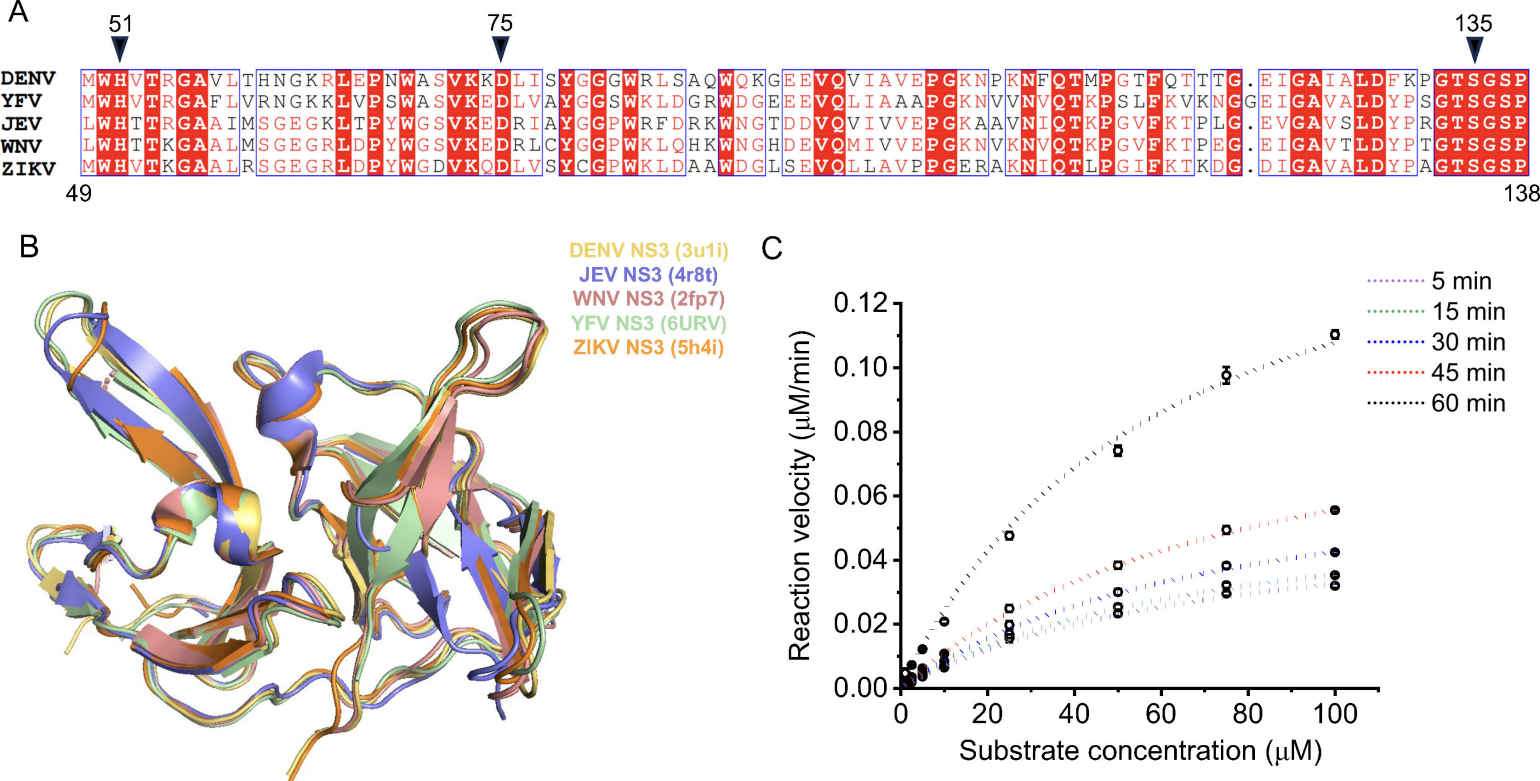
Conservation of the catalytic core region of NS3 protease and *in vitro* screening for anti-NS2B–NS3 protease activity. Catalytic triad residues are marked in black arrow. (**A**) Multiple sequence alignment of NS3 catalytic core domain of different flaviviruses, DENV (PDB ID: 3U1I), YFV (6URV), JEV (4R8T), WNV NS3 (2FP7), and ZIKV NS3 (5H4I). (**B**) Structural alignment of the NS3 protease of different flaviviruses showing high degree of conservation. (**C**) NS2B–NS3 enzyme kinetics at different time points using increasing concentrations of fluorogenic substrate.

The synthesized compounds were screened to assess their ability to inhibit the flaviviral protease NS2B–NS3 activity. NS2B–NS3 displays trypsin-like serine protease activity and plays a role in cleaving the flavivirus polyprotein at the NS2A-NS2B, NS2B–NS3, NS3-NS4A, and NS4B-NS5 junctions (38). All flavivirus (DENV, JEV, YFV, WNV, and ZIKV) NS2B–NS3 proteases were structurally conserved despite their low primary sequence homology (Fig. 3A). Additionally, the catalytic core contains entirely conserved residues, including His51, Asp75, and Ser135 (according to the DENV NS3 protease residue position), arranged in a catalytic triad with identical spatial organization (Fig. 3B). This configuration represents an optimal target for the development of broad-spectrum NS2B–NS3 protease inhibitors.

To assess the anti-DENV NS2B–NS3 activity of the compounds, we expressed the DENV2 NS2B–NS3 protease in BL21DE3 cells and subsequently purified it using Ni-NTA affinity chromatography, as outlined in Materials and Methods (39, 40). The protease activity assay was performed using different concentrations (1, 2.5, 5, 10, 25, 50, 75, and 100 µM) of a fluorogenic substrate (Abs-Nle-Lys-Arg-Arg-Ser-3-(NO_2_)Tyr) at various time points (5, 15, 30, 45, and 60 min), as described previously (39, 41). In our assay, the purified NS2B–NS3 protease showed a notable dose-dependent increase in reaction velocity after 1 h, with a less pronounced enhancement observed for shorter incubation times (Fig. 3C). Therefore, all subsequent assays were conducted with a 1 h incubation time. The optimized reaction was then replicated with different concentrations of compounds (**3a–3av**) to deduce the half maximal inhibitory concentration IC_50_ Of the 48 thiazole compounds tested, five compounds (**3u**, **3v**, **3aq**, **3au**, and **3at**) showed significant inhibition against DENV2 NS2B–NS3 protease with an IC_50_ value of less than 30 µM. Among them, compounds **3au** and **3aq** showed the most promising results, with an IC_50_ value of 20.76 and 15.15 µM respectively, compared to the dimethyl sulfoxide (DMSO) control (Tables 1 to 3).

**TABLE 1:** DENV2 NS2B–NS3 protease inhibitory activity for compounds **3a-3r***^a^*

		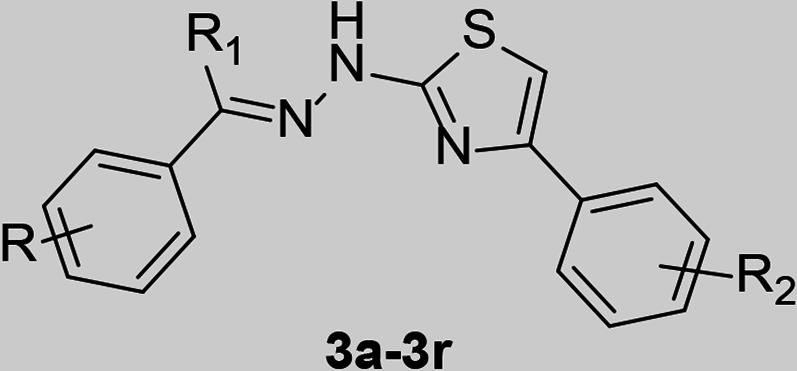		
Code	R	R_1_	R_2_	IC_50_ (µM)
3a	4-CH_3_	H	3-NO_2_	ND^*b*^
3b	3,5-(CH_3_)_2_	H	3-NO_2_	ND
3c	2-OCH_3_	H	3-NO_2_	229.1
3d	2,5-(OCH_3_)_2_	H	3-NO_2_	139.1
3e	3,4-(OCH_3_)_2_	H	3-NO_2_	502.4
3f	2-F	H	3-NO_2_	117.3
3g	3-Cl	H	3-NO_2_	298.9
3h	2-Br	H	3-NO_2_	342.1
3i	3-Br	H	3-NO_2_	413
3j	4-CH_3_	H	4-Cl	602.8
3k	3,5-(CH_3_)_2_	H	4-Cl	ND
3l	2-OCH_3_	H	4-Cl	ND
3m	2,5-(OCH_3_)_2_	H	4-Cl	215.6
3n	3,4-(OCH_3_)_2_	H	4-Cl	248.1
3o	2 F	H	4-Cl	ND
3p	3 Cl	H	4-Cl	ND
3q	2-Br	H	4-Cl	ND
3r	3-Br	H	4-Cl	ND

^*a*^ *In vitro* DENV2 NS2B/NS3pro inhibition assay of 48 synthesized thiazole compounds using a FRET substrate. The reaction mixture consisted of 5 µM substrate, 10nM NS2b-NS3pro in presence of different concentrations (1, 10 and 100 µM) of compounds, incubated for 1 h at RT. The fluorescent signal (emission at 460 nm upon excitation at 380 nm) of the cleaved substrate was recorded.

^*b*^ ND, no inhibition upon protease.

**TABLE 2:** DENV protease inhibitory activity for compounds **3s-3an***^a^*

		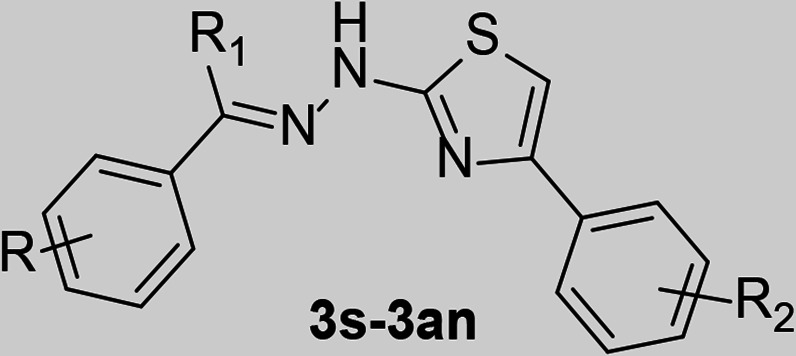		
Code	R	R_1_	R_2_	IC_50_ (µM)
3s	CH_3_	CH_3_	3-NO_2_	38.55
3t	4-CH_3_	CH_3_	3-NO_2_	113.2
3u	4-OH	CH_3_	3-NO_2_	28.66
3v	3,5-(OH)_2_	CH_3_	3-NO_2_	27.4
3w	2-OCH_3_	CH_3_	3-NO_2_	193.5
3x	3-OCH_3_	CH_3_	3-NO_2_	387.4
3y	4-OCH_3_	CH_3_	3-NO_2_	328.1
3z	2,4-(OCH_3_)_2_	CH_3_	3-NO_2_	170.7
3aa	2,5-(OCH_3_)_2_	CH_3_	3-NO_2_	ND^*b*^
3ab	3,4-(OCH_3_)_2_	CH_3_	3-NO_2_	31.38
3ac	4-Cl	CH_3_	3-NO_2_	ND
3ad	4-F	CH_3_	3-NO_2_	ND
3ae	4-CH_3_	CH_3_	4-Cl	ND
3af	4-OH	CH_3_	4-Cl	143.9
3ag	3,5-(OH)_2_	CH_3_	4-Cl	92.31
3ah	3-OCH_3_	CH_3_	4-Cl	262.5
3ai	4-OCH_3_	CH_3_	4-Cl	399.1
3aj	2,4-(OCH_3_)_2_	CH_3_	4-Cl	ND
3ak	2,5-(OCH_3_)_2_	CH_3_	4-Cl	124.3
3al	3,4-(OCH_3_)_2_	CH_3_	4-Cl	155.7
3m	4-Cl	CH_3_	4-Cl	ND
3an	4-F	CH_3_	4-Cl	ND

^*a*^ *In vitro* DENV2 NS2B/NS3pro inhibition assay of 48 synthesized thiazole compounds using a FRET substrate. The reaction mixture consisted of 5 µM substrate, 10 nM NS2b-NS3pro in the presence of different concentrations (1, 10, and 100 µM) of compounds, incubated for 1 h at RT. The fluorescent signal (emission at 460 nm upon excitation at 380 nm) of the cleaved substrate was recorded.

^*b*^ ND, no inhibition upon protease.

**TABLE 3:** DENV protease inhibitory activity for compounds **3ao–3av***^a^*

		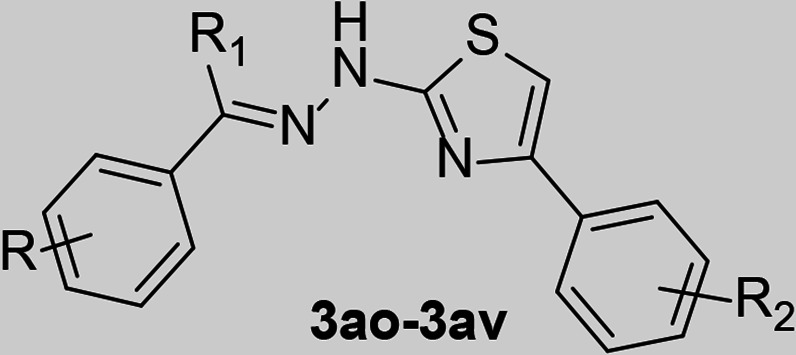		
Code	R	R_1_	R_2_	IC_50_ (µM)
3ao	4-OH	CH_3_	H	113.9
3ap	4-OH	CH_3_	4-CH_3_	55.99
**3aq**	4-OH	CH_3_	4-OCH_3_	15.15
3ar	4-OH	CH_3_	4-F	249.7
3as	3,5-(OH)_2_	CH_3_	H	ND^*b*^
3at	3,5-(OH)_2_	CH_3_	4-CH_3_	27.73
**3au**	3,5-(OH)_2_	CH_3_	4-OCH_3_	20.76
3av	3,5-(OH)_2_	CH_3_	4-F	37.15

^*a*^ *In vitro* DENV2 NS2B/NS3pro inhibition assay of 48 synthesized thiazole compounds using a FRET substrate. The reaction mixture consisted of 5 µM substrate, 10 nM NS2b-NS3pro in the presence of different concentrations (1, 10, and 100 µM) of compounds, incubated for 1 h at RT. The fluorescent signal (emission at 460 nm upon excitation at 380 nm) of the cleaved substrate was recorded.

^*b*^ ND, no inhibition upon protease.

### Structure**–**activity relationships (SAR)

To enable an SAR discussion, the data obtained from the *in vitro* screening are presented in Tables 1 to 3. The activity data for benzaldehyde-based thiazole derivatives (**3a–3r**) are presented in Table 1. For the 3-nitro derivatives (**3a–3i**), only **3d** and **3f,** featuring a 2,5-diOCH_3_ and 2-F substitutions, showed IC_50_ values of 139.1 and 117.3 µM, respectively. Among the 4-Cl derivatives (**3j–3r**), none of the compounds showed IC_50_ values less than 150 µM. It appears that electron-donating substitution (–OCH_3_) at the *ortho* (2) and *meta* (5) positions (**3d**) and -F substitution at the *ortho* (2) position (**3f**) in the 3-NO_2_ derivatives are more favorable than that in the 4-Cl derivative. However, other substitutions in both 3-NO_2_ and 4-Cl derivatives have shown IC_50_ value above 200 µM.

Among the thiazole derivatives of acetophenone origin (**3s–3an,** Table 2), we compared seven thiazole analogs of benzaldehyde origin (**3a**, **3c**, **3d**, **3e**, **3j**, **3m**, and **3n**) with those of acetophenone origin (**3t**, **3w**, **3aa**, **3ab**, **3ae**, **3ak**, and **3al**). In the substitution of -H with -CH_3_ at the R_1_ position, we observed significant improvement in potency in case of both 3-NO_2_ (**3a** and **3e**) and 4-Cl (**3ak** and **3al**) derivatives. An ~16-fold improvement was observed with **3ab** in comparison with **3e**. It is interesting to note that **3d** showing activity at 139.1 µM, completely lost its activity due to substitution of -H with -CH_3_ at the R_1_ position (**3aa**). Whereas, **3e** showing activity ~500 µM has shown dramatic increase in potency due to similar modification (**3ab**, 31.38 µM). This clearly shows that steric factors play a major role in accommodating the compound within the pocket with different binding conformation. This fact required to be checked with simulation studies. Compounds **3ak** and **3al** (4-Cl analogs of **3aa** and **3ab**, respectively) where found to be ~one fold better than their benzaldehyde counterparts, **3m** and **3n**. We expect no change in binding conformation with these compounds except improved hydrophobic interaction for compound **3ak** and **3al** due to -CH_3_ substitution at R_1_.

It is also observed that monomethoxy substituted derivatives (**3w–3y**) exhibited activity between ~200 and ~400 µM concentration. Whereas their dimethoxy analogs (**3z–3ab**) displayed distance dependent activity. When they are *ortho* to each other displayed the best activity (**3ab**, 31.38 µM), *meta* to each other displayed moderate activity (**3z**, 170.7 µM), and *para* to each other displayed no activity (**3aa**, ND). We expect favorable hydrogen bonding interaction(s) for compound **3ab** as reason for its better activity profile. Accordingly, compounds **3u** (28.66 µM) and **3v** (27.40 µM) displayed potency equivalent to compound **3ab** (31.38 µM) as both were having -OH functional group capable of establishing H-bonding interaction with target protein. A similar pattern was observed with 4-Cl counterparts (**3af–3al**) but are less potent.

Considering the activity of **3u** and **3v**, we retained the substitution at R and R_1_ and went on to vary the substitution at R_2_ (Table 3). Substitution at *para* position with methoxy group provided the potent compounds **3aq** and **3au** of the series with IC_50_ values of 15.15 and 20.76 µM, respectively. Methyl and Fluro are Grimm’s hydride isosteres with exactly opposite electronic characteristics. Activity profile of these derivatives displayed no significant change in potency with respect to 3,5-dihydroxy analogs (**3at** and **3av**) but exhibited >4-fold difference in case of 4-hydroxy analogues (**3ap** and **3ar**). This once again shows that rather than electronic parameter, steric factor plays a role, and accordingly, we expect a different binding orientation for **3aq** and **3au** within the binding pocket of DENV NS2B–NS3.

### Interaction profile of the antiviral compound with the catalytic core

**Fig 4:**
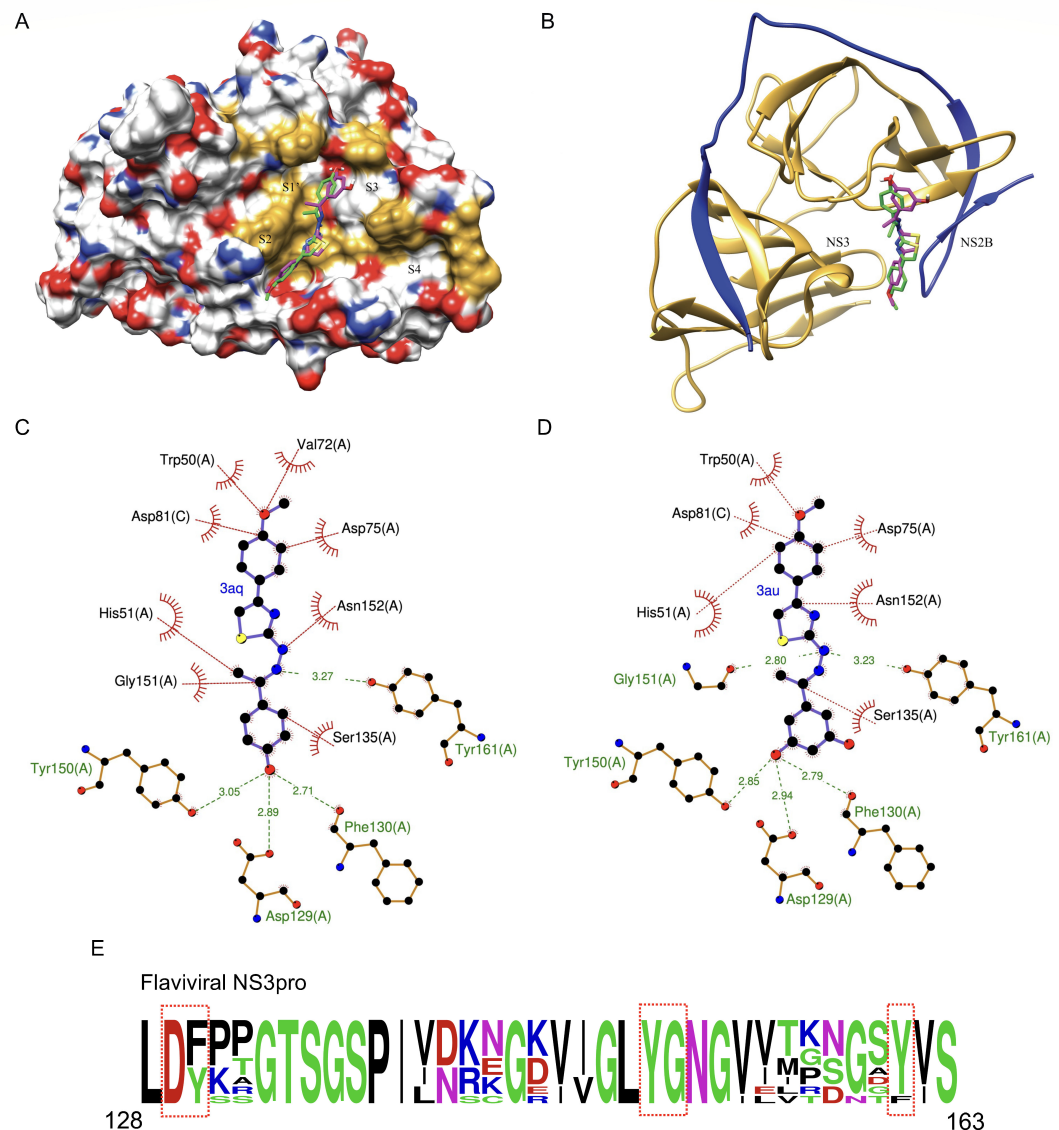
Docking image of compounds **3aq** (green) and **3au** (magenta) against DENV2 NS2B–NS3 protease crystal structure (PDB ID: 3U1I) (**A**) 3D representation of docking image in solvent-accessible surface colored by heteroatom while S1′, S2, S3, and S4 are the subpockets (residues lining the pockets colored golden). (**B**) The ribbon model depicts the protease accommodating ligands, aligned as shown in (**A**) NS3 was colored golden, and NS2B was colored blue. Images were generated using Chimera software. (**C and D**) Two-dimensional interaction diagrams illustrating the interactions of compounds **3aq** and **3au** with DENV2 NS2B–NS3 protease (PDB: 3U1I). (**E**) Logo plot showcasing amino acid frequencies in NS3 protease across various flaviviruses, emphasizing the conservation of crucial interacting residues: D129, F130, Y150, G151, and Y161.

To elucidate the crucial interactions underlying DENV protease inhibitory activity, binding mode analysis was conducted for the top two compounds (**3aq** and **3au**). Structure of active closed NS2B–NS3 complexof DENV3 (PDB: 3U1I) (42) was used for the interaction study (Fig. 4A B; Table 4). This structure represents the closed, active conformation that is essential for inhibitor binding, which is more relevant for drug design compared with open, inactive conformation available for DENV2 (43). To mimic the binding pocket of DENV2, the following residues were mutated: V36I, T115L, and K157R. Both the compounds, **3aq** and **3au** accommodated within the pocket in a similar fashion and displayed interactions that are common for both (Fig. 4A B). Figure 3C and D displays a 2D interaction plot for compounds **3aq** and **3au** with DENV NS2B–NS3 protease. Hydroxy functional group [4-OH and 3,5-(OH)_2_] on hydrazone phenyl ring established three H-bonding interaction with sidechain carboxylic acid group of ASP129 (S3), backbone carbonyl oxygen of PHE130, and sidechain phenolic hydroxyl group of TYR150 (S1). Anchoring of hydroxyl functional group of **3aq** and **3au** due to these H-bonds facilitate hydrophobic interaction of phenyl ring and R1 methyl group with S1′ (HIS51, SER135, and GLY151) and S3 (ASP129) subpocket residues. Both S1, S1′, and S3 were not occupied, rather the peripheral region (entrance) shows interaction with **3aq** and **3au**. Similar interaction is observed for substrate peptide backbone as sidechains occupy the subpockets. An additional H-bonding interaction was observed between sidechain phenolic hydroxyl group of TYR161 with hydrazone’s imino nitrogen of **3aq**. In case of **3au**, presence of dihydroxy functional group slightly pushed the compound that facilitated establishment of two additional H-bonding interaction for **3au**. One between phenolic hydroxyl group of TYR161 and hydrazone’s amino nitrogen of **3au**, and other between backbone carbonyl oxygen of GLY151 and hydrazone’s amino hydrogen of **3au**. Anchoring due to these additional H-bonding interactions favored hydrophobic interaction of 4-methoxy phenyl ring at the 4th position of the thiazole ring with S2 (ASP75) subpocket residue along with NS2B ASP81 residue shaping the S2 pocket in a closed active conformation. Interaction of both the phenyl rings thus kept the thiazole ring with hyradrazone linker showing hydrophobic interaction with S2 (ASN152) subpocket residues. S1 and S2 subpockets demand dibasic amino acid requirement that poses challenge in optimizing the PK profile of peptidic inhibitor design. Our compounds did not show any interaction with residues lining S1 subpocket (LEU115, SER163, and ILE165) except TYR150 that tend to show electrostatic interaction with arginine/lysine (P1) of substrate peptide, a dibasic requirement. In our case, electrostatic interaction was fulfilled by the H-bonding interaction with phenolic -OH of hydrazone segment. While the basic requirement for S2 subpocket has been compensated by hydrophobic interactions between thiazole–hydrazone segment and HIS51, ASP75, and ASN152 (S2) (Fig. 4E). Additionally, analysis of protein–ligand complexes with the PLIP server (https://plip-tool.biotec.tu-dresden.de/) (44, 45) revealed π–π interaction between phenyl rings of hydrazone segment and TYR161 for both the compounds. Further, in the case of compound **3u** the imidazole ring of catalytic HIS51 residue exhibited both parallel and T-shaped π–π interactions with phenyl ring at 4th position of thiazole and thiazole ring itself, respectively. As expected, the phenyl hydrazone segment showed interactions with residues lining the S1 and S2 subpockets (Fig. 1), as well as residues lining the S1′ and S3 subpockets, which justifies their observed inhibitory activity in-vitro. The smaller size and relatively linear structure of the compounds facilitated their orientation, allowing the 4-phenylthiazole segment to interact effectively with residues lining the S2 subpocket. Notably, the compounds displayed interactions with residues that are largely conserved not only for DENV serotypes but also for other flaviviruses (Fig. 4E; Table 4) suggesting their broad-spectrum antiviral potential against different members of the Flaviviridae family.

**TABLE 4:** Protein–ligand interaction: interacting residues and type of interactions along with conservation nature across different serotypes of dengue and different types of flavivirus^*a*^

Residue	Hydrophobic interaction		H-bonding interactions		π–π interaction		Conservation	
	3aq	3au	3aq	3au	3aq	3au	DENV	Flavivirus
TRP50	Y	Y	N	N	N	N	99.98%	99.91%
HIS51 (S1′, S2)	Y	Y	N	N	N	Y	100%	100%
VAL72	Y	N	N	N	N	N	99.90%	99.92%
ASP75 (S2)	Y	Y	N	N	N	N	99.98%	99.97%
ASP81 (NS2B)	Y	Y	N	N	N	N	100%	92.90%
ASP129 (S3)	Y	Y	Y	Y	N	N	99.98%	99.99%
PHE130	Y	Y	Y	Y	N	N	100%	84.33%
SER135 (S1′)	Y	Y	N	N	N	N	99.98%	99.98%
TYR150 (S1)	Y	Y	Y	Y	N	N	100%	99.98%
GLY151 (S1′)	Y	Y	N	Y	N	N	100%	100%
ASN152 (S2)	Y	Y	N	N	N	N	99.98%	99.99%
TYR161	Y	Y	Y	Y	N	Y	100%	97%

^*a*^ Y, interacting; N, not interacting. Subpocket information is provided in parentheses.

### Detailed characterization of the inhibitory action of two potent compounds

**Fig 5:**
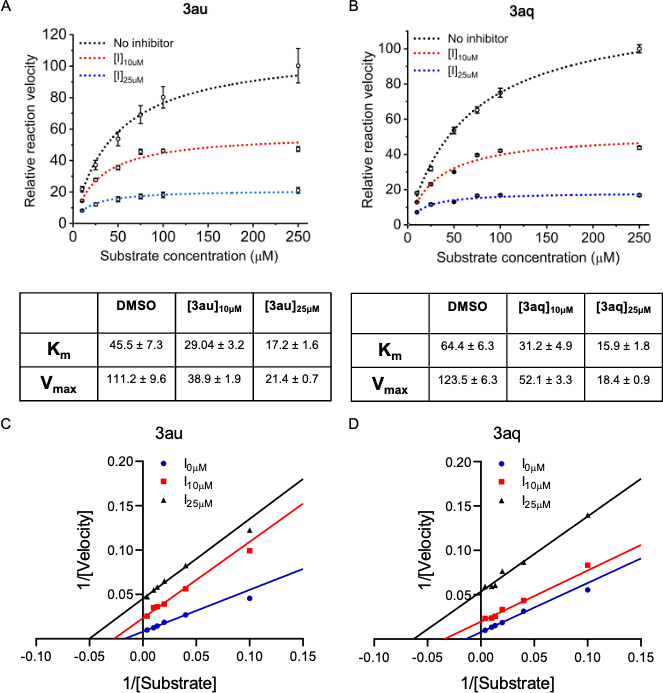
Characterization of the inhibition pattern of **3au** and **3aq**. (**A and B**) *In vitro* protease inhibition assay was performed in presence of two different concentrations of each compound using increasing substrate concentrations. NS2B–NS3 enzyme kinetics was determined by fitting the nonlinear equation in Origin2023b software. Table representing two kinetics parameters, K_m_ and V_max_ of the control enzyme as well as **3au** and **3aq**-treated enzyme. The kinetic data were fitted into the Michaelis–Menten equation. (**C and D**) Furthermore, Lineweaver–Burk plot was done in Origin2023b. Each experiment is performed in biological triplicate and repeated thrice.

As **3au** and **3aq** compounds emerged as the two most promising candidates, we characterized their NS2B–NS3 inhibitory activity in greater detail using the previously mentioned DENV NS2B–NS3 protease assay (Fig. 3D) (39, 41). Experiments were performed to determine the kinetic parameters, inhibition constants, and inhibition mechanisms of these compounds. Enzymatic activity and corresponding reaction velocity were measured with various substrate concentrations either in presence of the vehicle control or two different concentrations of compounds. Several inhibitory mechanisms, including competitive, uncompetitive, and mixed/non-competitive mechanisms, were explored for each compound. As shown in Tables A and B in Fig. 5, the presence of the compounds reduced both the K_m_ and V_max_ of the reaction, thereby indicating an uncompetitive mode of inhibition for both compounds. The double reciprocal plot (Lineweaver–Burk plot) ( Fig. 5C and D) also validates the mode of enzymatic inhibition against DENV NS2B–NS3 protease. This is also supported by the docking analysis, which showed no polar interaction with the triad site (Fig. 4).

### Anti-flaviviral activity of 3aq and 3au

*In vitro* studies suggested that **3au** and **3aq** can inhibit DENV NS2B–NS3 activity (Fig. 2 to 4). Hence, we aimed to assess the antiviral activity of the compounds. First, cytotoxic impact of these compounds was evaluated using MTT assay. Our data demonstrated that both compounds showed moderate cytotoxicity as determined through 50% cytotoxic concentrations (CC_50_) of **3au** and **3aq** in BHK-21 cells. Both compounds are non-toxic up to 25 µM concentration with a CC_50_ of 76.62 ± 2.74 µM and 82.03 ± 2.38 µM for **3au** and **3aq**, respectively. Subsequently, we evaluated the effectiveness of these compounds in inhibiting DENV2 replication. BHK-21 cells were infected with the virus in presence of increasing concentration of these compounds, and harvested at 24 hpi. The virus replication rate was assessed by quantifying the copy number of viral genomic RNA using quantitative real-time PCR (qRT-PCR). Our data suggested that both **3au** and **3aq** exhibited significant reduction in viral RNA load, thereby establishing their inhibitory effect upon virus replication. As evidenced in Fig. 5A and B, **3au** and **3aq** reduced virus replication in a dose-dependent manner with 50% inhibitory concentration (IC_50_) of 7.38 ± 0.60 µM and 4.47 ± 0.41 µM, respectively.

**Fig 6:**
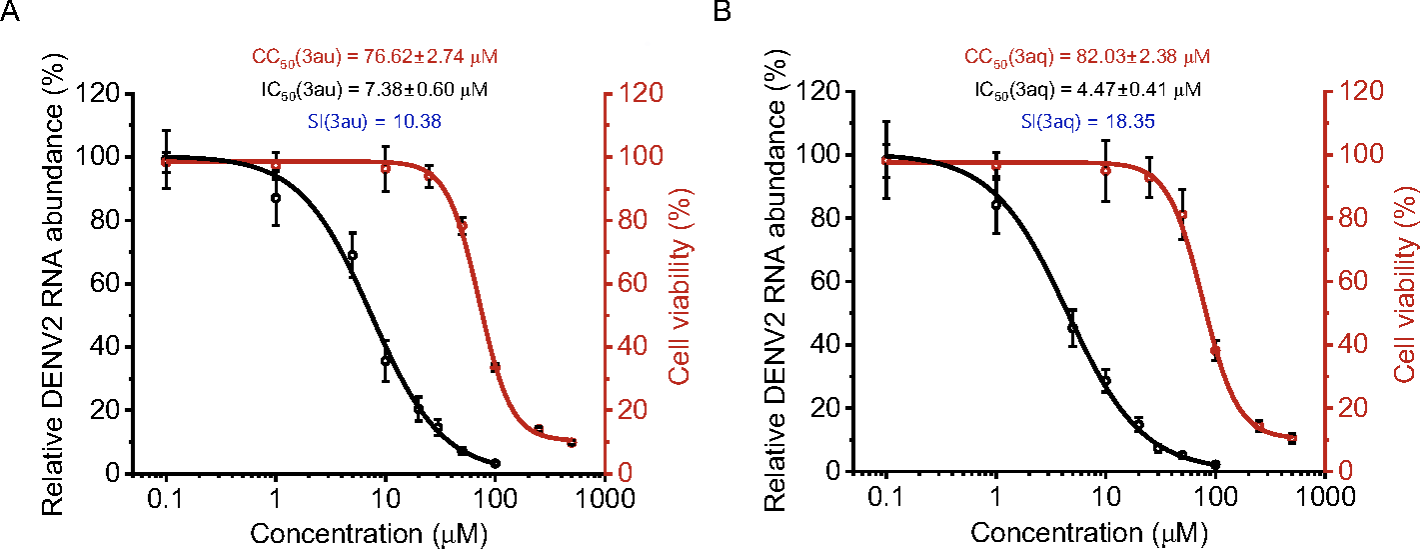
Anti-viral activity of compounds against DENV2. BHK-21 cells were treated with different concentrations of the compounds (**3aq** and **3au**) for 24 h. MTT assay was performed to determine cytotoxicity of the compounds **3au** and **3aq** (A, B; red). BHK-21 cells were infected with DENV2 (MOI: 0.1) in the presence of different concentrations of each compound and incubated for 24 h. Reverse transcription and subsequent quantitative RT-PCR (qRT-PCR) were performed to measure virus replication. The data are expressed as fold changes of the viral RNA levels normalized to GAPDH control relative to the corresponding DMSO-treated sample (A, B; black). Further, CC_50_ and IC_50_ of the compounds were determined by fitting the nonlinear equation in Origin2023b software. SI, selectivity index (CC_50_/IC_50_). Each experiment is performed in biological triplicate and repeated thrice.

Docking analysis revealed that two compounds preferentially bind to several residues within and around the catalytic core, most of which are conserved across different flavivirus NS2B–NS3 proteases (Fig. 4E) (19). Therefore, we sought to evaluate the efficacy of these compounds against JEV to assess their broad-spectrum anti-viral action against flaviviruses. Both the compounds showed strong antiviral effects against JEV, with an IC_50_ of 1.16 ± 0.16 µM and 1.03 ± 0.21 µM for **3au** and **3aq**, respectively (Fig. 6A and B). Interestingly, both the compounds showed higher selectivity index against JEV (67.8 and 82.03 for **3au** and **3aq**, respectively) than against DENV2 (10.38 and 18.35 for **3au** and **3aq**, respectively), which indicates more pronounced antiviral effect of these compounds towards JEV.

**Fig 7:**
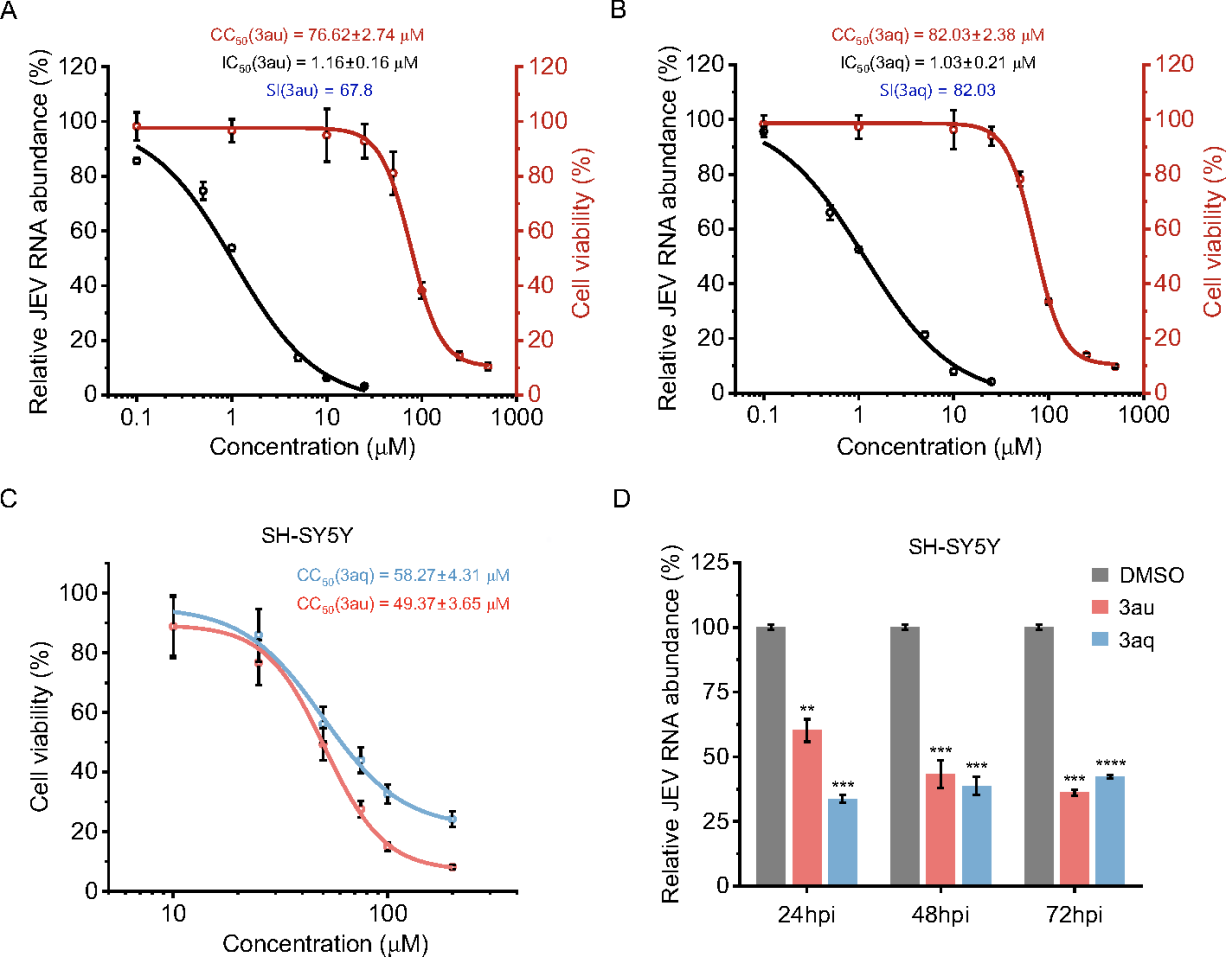
Anti-viral activity of compounds against JEV. (**A and B**) BHK-21 cells were infected with JEV (MOI: 0.1) in the presence of different concentrations of each compound and incubated for 24 h. Reverse transcription and subsequent quantitative RT-PCR (qRT-PCR) were performed as described previously to quantify the reduction in viral RNA load (black). Cellular cytotoxicity was measured by MTT assay (red). (**C**) Cellular cytotoxicity was measured by MTT assay for **3au** and **3aq** in SH-SY5Y cells. SH-SY5Y cells were infected with JEV virus (MOI = 0.1) for 24, 48, and 72 h with or without 10 µM of compounds, and the antiviral activity was assessed using qRT-PCR based method (**D**). Each experiment is performed in biological triplicate and repeated thrice. CC_50_ and IC_50_ of the compounds were determined by fitting the nonlinear equation in Origin2023b software. SI, selectivity index (CC_50_/IC_50_). Statistical significance was analyzed by two-tailed equal variance Student’s *t*-test: *P < 0.05, **P < 0.01, ***P < 0.001, and ****P < 0.0001.

We further studied the effect of the compounds on JEV replication in the human neuroblastoma SH-SY5Y cells, which represents a suitable model cell line for a neuropathic virus, like JEV. At first, the cytotoxicity was studied for both compounds in SH-SY5Y cells. As indicated in the Fig. 7C, **3au** and **3aq** displayed CC_50_ of 49.37 ± 3.65 and 58.27 ±  4.31 µM, respectively. Then, the SH-SY5Y cells were infected with the JEV virus in the absence or presence of **3aq** or **3au** (10 µM), and viral genomic RNA copies were quantified using qRT-PCR at 24, 48, and 72 hpi, as described previously. Results demonstrated that **3au** and **3aq** treatments caused a sharp reduction in viral RNA levels at different time points, with 67%–58% reduction for **3au** and 64%–40% reduction for **3aq**, at different times of post infection (Fig. 7D). Together, these studies indicated that **3au** and **3aq** significantly inhibit the replication of both DENV2 and JEV, suggesting their potential as lead candidates for the development of antiviral therapies targeting flavivirus infections.

**Fig 8:**
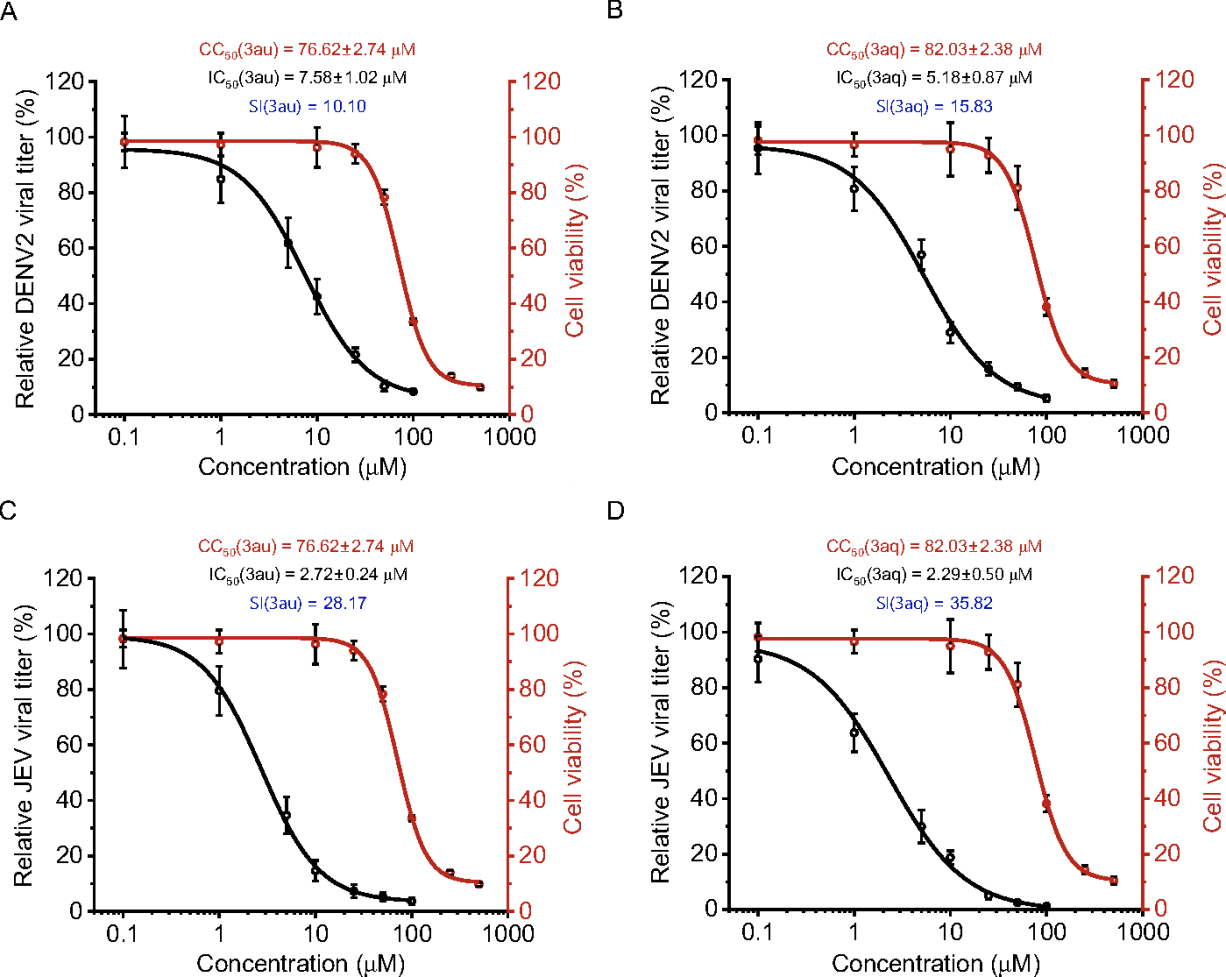
BHK-21 cells were infected with DENV2 (**A and B**) and JEV (**C and D**) respectively in the presence of different concentrations of each compound at a multiplicity of infection (MOI) 0.1 and incubated for 24 h at 37⁰C. Viral titer in the supernatant of infected cells was measured using TCID_50_. Further, CC_50_ and IC_50_ of the compounds were determined by fitting the nonlinear equation in Origin2023b software. SI, selectivity index (CC_50_/IC_50_). Each experiment is performed in biological triplicate and repeated thrice.

To this end, we assessed the antiviral activity of shortlisted compounds by evaluating their ability to inhibit progeny virus production. BHK-21 cells were infected with DENV2 and JEV (MOI: 0.1) in the presence of different concentrations of **3aq** or **3au** or with DMSO, and viral titer was determined in the supernatants harvested at 18 hpi. Compound **3aq** caused the maximum reduction of viral titer showing TCID_50_ of 5.18 ± 0.87 and 2.29 ± 0.50 µM against DENV2 and JEV, respectively (Fig. 8B and D). Then, **3au** exhibited TCID_50_ of 7.58 ± 1.02 and 2.72 ± 0.24 µM against DENV2 and JEV, respectively ( Fig. 8A and C). These data strongly support the efficacy of the compounds in suppressing JEV and DENV2 infection.

### Compounds 3aq and 3au impact viral replication and accumulation of viral genomic RNA at late stages of infection

**Fig 9:**
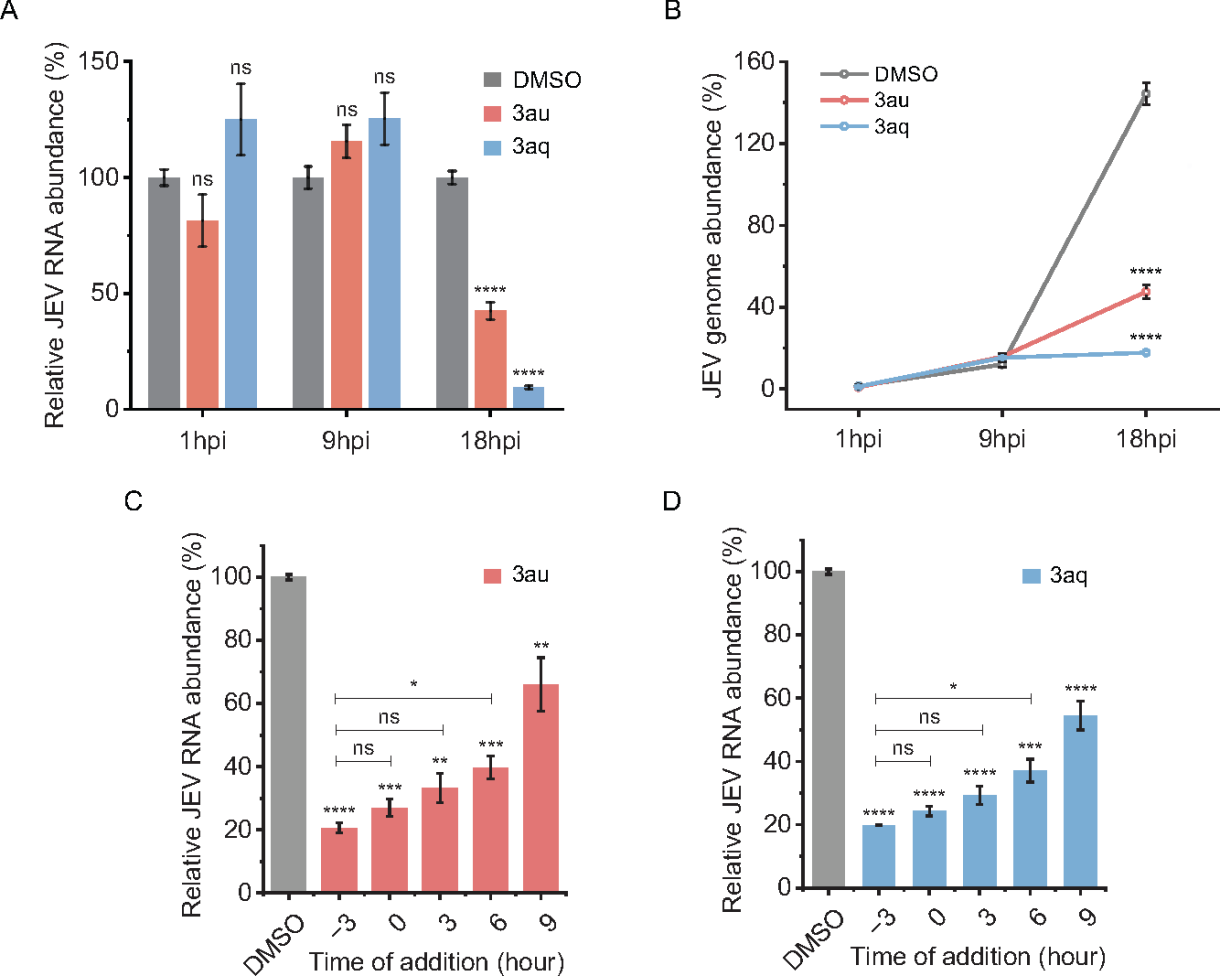
Detailed molecular mechanism of the inhibitory action of the compounds. (**A and B**) BHK-21 cells were infected with JEV virus (MOI = 0.1) for 1, 9, and 18 h with or without 10 µM of compounds. Total RNA extracted at indicated times was evaluated by qRT-PCR. The data are expressed as fold changes of the viral RNA levels normalized to GAPDH control relative to the corresponding DMSO treated samples at each time point. (**B**) Same experiment as in (**A**) but expressed as relative to the initial inoculation amount (at 1 hpi). BHK-21 cells were infected with JEV (MOI: 0.1) and treated with **3au** (**C**) and **3aq** (**D**) pre + during + post (−3), during + post (0), and post (3, 6, 9) infection. Viral RNA load were assessed at 24 hpi using qRT-PCR. The data are expressed as fold changes of the viral RNA levels normalized to GAPDH control relative to the corresponding DMSO-treated sample. Each experiment is performed in biological triplicate and repeated thrice. Statistical significance was analyzed by two-tailed equal variance Student’s *t*-test: *P < 0.05, **P < 0.01, ***P < 0.001, and ****P < 0.0001.

Flaviviral replication kinetics showed delayed accumulation of viral RNA at later time points of infection cycle (43). This is because the initial translation of the viral RNA genome leads to the production of a viral polyprotein, which upon processing by the NS2B–NS3 protease, leads to the generation of viral RdRp (NS5) and other NS proteins necessary for RNA synthesis (15). Thus, inhibition of the NS2B–NS3 protease by the two potential compounds should affect viral genomic RNA accumulation within infected cells only at later time points post-infection (46). To validate this hypothesis, we assessed the effect of the compounds upon dynamic accumulation of viral RNA at different times of post-infection. Cells were infected with the virus in the absence or presence of **3aq** or **3au** (10 µM) and harvested at different time points post-infection, and viral genomic RNA copies were estimated using qRT-PCR. Figure 9A shows the relative levels of viral genomic RNA in treated versus control sets, and Fig. 9B shows the absolute expression of viral RNA in the control and treated sets during the course of infection. As expected, the abundance of viral genomic RNA remained low at the early time points of infection (1 and 9 hpi) and subsequently peaked at 18 hpi (Fig. 9B). Interestingly, both compounds showed a drastic effect on viral genomic RNA abundance only at the later time point post-infection (18 hpi), whereas no effect was observed at the early time points (1 and 9 hpi) (Fig. 9A and B). These data clearly demonstrate that **3aq** and **3au** exert their antiviral effects by blocking genomic RNA replication at a later stage of infection, which is in line with previous findings. This is an indirect effect of NS2B–NS3 protease inhibition, which in turn blocks polyprotein processing and assembly of the viral RNA synthesis machinery.

One of the criteria of a potential compound to be effective antiviral is to show its efficacy to reduce viral load and hence disease burden when applied post initiation of infection. Considering the mode of action of the compounds **3au** and **3aq**, they should satisfy this criteria. To evaluate this potential, time of addition experiment was conducted for both compounds to inhibit JEV infection. BHK-21 cells and the virus were pre-treated for 3 h with 10 µM of compounds followed by infection and subsequent incubation in presence of the same (pre + during + post). Alternatively, the compounds (10 µM) were added either during the infection (during + post) or at different times post-infection as indicated (Fig. 9C and D). Virus amplification was measured at 24 hpi by measuring viral RNA accumulation as described above. Results (Fig. 9C and D) demonstrate that both compounds significantly inhibited viral replication even when added at 9 hpi. This sustained inhibition indicates a potential therapeutic window for intervention, highlighting the compounds' ability to impede viral replication after initial infection. Additionally, there were no differences in viral RNA accumulation between the sets where compounds were added either 3 h pre- or post-infection, thereby confirming that the compounds did not affect any of the steps during viral entry but rather blocked one of the post entry replication steps as inferred earlier. Together, our data not only established the antiviral activity of the compounds against DENV2 and JEV but also pointed out the mechanism of action of these compounds during the specific steps of the virus life cycle.

### Conclusion

With the increasing impact of flavivirus infections worldwide, a broad-spectrum antiviral agent targeting the highly conserved viral machinery is need of the hour. The proteolytic processing of viral polyproteins is a key event that is imperative for the production of viral structural and non-structural proteins. This ensures the smooth occurrence of the subsequent steps of the viral life cycle, including replication of genomic RNA, their packaging into newly assembled viral capsids, and the final release of mature virion particles from the host cells (17). Therefore, virus-encoded proteases have emerged as effective antiviral targets not only for flaviviruses but also for other positive-sense RNA viruses, such as picornaviruses or coronaviruses (47). Interestingly, the catalytic core (catalytic triad) of the flavivirus NS3 protease (DENV, JEV, YFV, WNV, and ZIKV) shows high sequence and structural conservation, with similar sub-pocket residues and substrate amino acid preferences (at the P1 and P2 positions) (18, 19). Considering these factors, a previously reported retro-tripeptide (33) was transformed into a thiazole heterocyclic template and derivatized to synthesize a series of thiazole compounds (**3a–3av**). These compounds were evaluated for their DENV2 NS2B–NS3 protease inhibitory activities using a high-throughput *in vitro* screening system (39–41). The top two compounds (**3aq** and **3au**) showing the highest inhibition in the *in vitro* screening were shortlisted for further characterization. Docking of these two compounds on the mutated (to mimic DENV2) DENV-3 NS2B–NS3 protease crystal structure (PDB ID: 3U1I) revealed that these compounds did interact with the catalytic triad (Fig. 3), rather interacted with residues situated within subpockets S1′, S2, and S3 and are conserved across different flaviruses (Fig. 4C and D). Interestingly, both compounds affected K_m_ and V_max_, implying uncompetitive inhibition of the NS2B–NS3 protease (Fig. 5).

The compounds **3au** and **3aq** showed effective inhibition of DENV2 in BHK-21 cells as evidenced through significant reduction in viral RNA load in a dose dependent manner. The IC_50_ value of the compounds were in the low micromolar range (IC_50_ 7.38 ± 0.60 and 4.47 ± 0.41 µM for **3au** and **3aq**, respectively). Interestingly, the residues targeted by both the compounds remained conserved across a wide range of flaviviruses, including DENV, JEV, YFV, WNV, and ZIKV, indicating a potential broad-spectrum antiviral activity against these viruses (Fig. 4E; Table 4). Supporting this notion, the compounds also served as a potent inhibitor of JEV, with even lower IC_50_ values of 1.16 ± 0.16 and 1.03 ± 0.21 µM for **3au** and **3aq**, respectively. Both of these compounds showed low to moderate cytotoxicity in their effective range thereby achieving high selectivity index, specifically against JEV infection. Additionally, the compounds showed promising effects by inhibiting viral amplification as evidenced though TCID_50_ assay (Fig. 7A-D), and the inhibition of viral titer can be well correlated with the reduction of viral RNA synthesis, entrenching the drastic effect of these compounds upon viral infection cycle. Both compounds again seem to be more effective towards JEV than DENV2.

SH-SY5Y cells are extensively used as a neuronal-like *in vitro* model for studying neuropathology during JEV or other flaviviral infections. This is why SH-SY5Y cells contributed to significant biological and pharmacological breakthroughs (48–51). Study in human neuroblastoma SH-SY5Y cells also established the antiviral effects of these compounds against JEV. (Fig. 7D)

We also determined the inhibitory mechanisms of the shortlisted compounds on viral life cycle. The time of addition experiment highlighted the action of these compounds, particularly upon viral replication steps. Furthermore, replication kinetics experiments showed that the rate of accumulation of viral genomic RNA by both these compounds remained unchanged at early time points (1 and 9 hpi) but was significantly reduced at later time points post-infection (18 hpi). These data together confirm that the shortlisted compounds do not affect early events of the infectious cycle, such as receptor binding, endocytosis, uncoating, or release of viral genomic RNA within the host cellular cytoplasm. Rather, the effect of these compounds upon the virus life cycle correlates with their NS2B–NS3 inhibitory activity, which affects polyprotein processing and eventually blocks viral RNA synthesis. Together, these data establish the newly synthesized **3aq** and **3au** compounds as inhibitors of DENV2 NS2B–NS3 protease, which has the potential to be developed as antivirals agents against multiple members of the Flaviviridae family.

## MATERIALS AND METHODS

### General comments

All chemicals and reagents were purchased from commercial suppliers (CDH/Merck/Sigma-Aldrich/Alfa Aesar/Rankem) and were used without purification. TLC plates (Merck) were used to monitor the completion of the reactions in either iodine or UV chamber (at 254 nm). Melting points were determined using OPTIMELT (Stanford Research System, USA) and were uncorrected. All the final compounds and a few intermediates were characterized by ^1^H-NMR and ^13^C-NMR spectroscopy on a JEOL JNM-ECZ 400S/L1400 MHz NMR spectrometer with tetramethylsilane (TMS) as the internal standard, and CDCl_3_ and DMSO-d_6_ were used as solvents. For the^1^H-NMR spectra, the coupling constant (J) is expressed in Hertz (Hz). The chemical shifts (δ) of NMR have been reported in parts per million (ppm) units relative to TMS. The splitting patterns are abbreviated as singlet (s), doublet (d), double doublet (dd), triplet (t), quartet (q), and multiplet (m). Mass spectra were recorded on a Thermo Scientific Ultimate 3000/Waters (Milford, USA) using electron spray ionization (ESI). The molecular docking studies were performed on MacBook Pro with Apple M2 chip and 8 GB Memory running on macOS Sonoma 14.6.1. ChemDraw 19.1 (Perkin-Elmer Informatics) was used to draw ligand structures. The protein structure was downloaded from the Protein Data Bank (https://www.rcsb.org/) (52). LigPlot+ was used for visualization of the 2D ligand interactions (53).

### General procedure for the synthesis of (E)-2-benzylidene /(1-phenylethylidene)-hydrazine-1- carbothioamides (2a–u)

To a solution of acetophenone/benzaldehyde (0.01 M) and thiosemicarbazide (0.012 M) in methanol (25 mL), a catalytic amount of acetic acid glacial (0.6–0.9 mL) was added. The mixture was stirred for a period of 2–4 h at room temperature. After confirming the completion of the reaction by TLC, the solution was diluted with ice water (50 mL), and the obtained solid was filtered, washed with distilled water, dried, and recrystallized from hot methanol.

### General procedure for the synthesis of (E)-2-(2-benzylidene/(1-phenylethylidene)-hydrazineyl]-4-phenyl-1,3-thiazoles (3a–3av)

An appropriate amount of phenacyl bromide (0.01 M) was then added to a solution of **2a**-**u** (0.01 M) in methanol (25 mL). The mixture was stirred for a period 30–90 min at room temperature. The completion of the reaction was confirmed by TLC and further the solution was diluted with ice water (50 mL), and the obtained solid was filtered, washed with distilled water, dried, and recrystallized from hot methanol.

### Cells and viruses

Baby hamster kidney fibroblast (BHK-21 cell line) and SH-SY5Y (neuroblastoma cell line) cells were maintained in Dulbecco’s modified Eagle’s medium (DMEM) supplemented with 10% fetal bovine serum (FBS) and penicillin and streptomycin antibiotics (Gibco) at 37 °C in 5% CO_2_. *JEV* strain Vellore P20778, and *DENV2* strain IND_P23085_1960 was used to infect BHK-21 cells.

### DENV2 NS2B–NS3 protease expression and purification

The pET28a plasmid encoding the NS2B/NS3 protease sequence from DENV2 (pET28a-DENV2-NS2B-4GS4G-NS3) (54) was transformed into *Escherichia coli* BL21(DE3) cells. BL21(DE3) cells containing DENV2 NS2B–NS3 protease expression plasmids were grown in Luria–Bertani (LB) medium containing 50 µg/mL kanamycin at 37 °C until OD_600_ reached 0.6. First, the cultures were incubated for 2–3 h at 37 °C until an OD_600_ of 0.6–0.8 was reached. Isopropyl β-d-1-thiogalactopyranoside (IPTG) was added at a final concentration of 1 mM. The cells were incubated for 5 h at 30 °C with shaking at 100 rpm to induce protein expression.

The cells were harvested by centrifugation at 4,500 rpm (REMI NEYA16 centrifuge) for 15 min at 4 °C, and the pellets were frozen in liquid nitrogen and stored at −80 °C. The cell pellets were thawed and completely resuspended in lysis buffer (25 mM Tris pH 7.9, 0.5 M NaCl, 5 mM imidazole, 5% glycerol). After resuspension, the solution was maintained on ice. For purification, the cells were lyzed by sonication using an Ultrasonic Cell Disruptor. The lysate was centrifuged at 15,000×*g* for 40 min at 4 °C. Prior to Ni^2+^ affinity chromatography, DNAse (Sigma-Aldrich) was added to the supernatant. The soluble 6-His-NS2B–NS3 protease in its native form was filtered, batch-bound to 2 mL Ni^2+^-NTA (Qiagen) resin (pre-equilibrated with column buffer), and incubated for 1 h at 4 °C. The resin was removed from the unbound fraction by centrifugation, and the resin containing the bound protein was collected and loaded into columns (Bio-Rad). The packed column was washed extensively with 10 mL wash buffer containing 5, 10, and 50 mM imidazole. The recombinant protein was eluted using elution buffer (lysis buffer containing 250 mM imidazole). The purified protein was analyzed using 12% SDS-PAGE. The fractions with the highest amount of pure protein were desalted and concentrated with a centrifugal concentrator (Sartorius: 10 kDa MWCO) and then washed 4–5 times with 40 mL of storage buffer (100 mM Tris, pH 7.9, 50 mM NaCl, and 50% glycerol) at 4 °C to remove all imidazole. The protein was distributed into 50 µL aliquots and frozen in liquid nitrogen for storage at −80 °C for further use in dengue protease activity and inhibition studies.

### NS2B–NS3 protease assay and steady-state kinetics

The selected compounds were analyzed using *in vitro* protease assays performed in black 96-well plates. Standard reaction mixtures (60 µL) containing 50 mM Tris·HCl (pH 8.5), 1 mM CHAPS, 20% glycerol, 10 nM DENV2 NS2B–NS3 protease, 10 µM inhibitor (dissolved in DMSO) or DMSO, and 10.0 µM tetra-peptide substrate trifluoroacetate-Nle-Lys-Arg-Arg-AMC (ALFA Chemicals) were added to the reaction mixture and incubated for 1 h at room temperature. All assays were performed in triplicate. The release of free AMC was measured using a Varioskan LUX multimode microplate reader (Thermo Fisher Scientific) at excitation and emission wavelengths of 380 and 460 nm, respectively. Control fluorescence values obtained in the absence of inhibitors were considered 100%, and those obtained in the presence of inhibitors were calculated as the percentage of inhibition of the control using Microsoft Excel and plotted using Origin Pro 2023b. The background AMC in the absence of protease was subtracted before data analysis. Further, protease assay was done using different concentration of the compounds (1, 10, and 100 µM) to evaluate the IC_50_ for the *in vitro* assay. IC_50_ curves were fitted and determined by fitting the nonlinear equation in Origin2023b software. A similar approach was used to analyze the enzymatic kinetics of protease inhibition. The reaction progress was monitored by the release of free aminomethylcoumarin (AMC), as previously described. The substrate was incubated with or without the inhibitor in the aforementioned reaction buffer in the presence of varying concentrations of the substrate. The reactions were initiated by the addition of the NS2B–NS3 protease. Protease activity was monitored for 1 h based on the initial rate of increase in fluorescence intensity at 460 nm, with an excitation wavelength of 380 nm. The results were analyzed using Origin Pro 2023b and the Michaelis–Menten equation to obtain the apparent Michaelis–Menten constants and maximal velocities. All assays were performed in triplicate. For each tested compound, two different concentrations of the inhibitor (0, 10, and 25 µM) were used at various concentrations (0–250 μM) of the substrate. The mechanism of inhibition was determined by observing the deviations in the apparent K_m_ and V_max_ values.

### MTT assay

The MTT assay was performed to determine cellular metabolic activity as an indicator of cell viability, proliferation, and cytotoxicity. Thus, to determine whether these compounds had any toxic effects on cellular viability, an MTT assay was performed following the protocol described previously by Mosmann (1983) (55, 56). Briefly, BHK-21 and SH-SHY5Y cells were seeded into 96-well plates at a density of 20,000 cells/well. Almost 16–18 h later, the cells were treated with different concentrations of the compounds (**3au** and **3aq**) for 24 h at 37 °C in 5% CO_2_. Thereafter, the medium was aspirated, and 100 µL of MTT reagent (5 mg/mL, SRL) in PBS was added to the cells and incubated for 3 h at 37 °C. Formazan crystals were formed in each well and resuspended in 100 µL of dimethyl sulfoxide (DMSO) (Sigma, USA) at room temperature for 20 min to completely dissolve the crystals. The absorbance of the solution in a 96-well plate was measured at 595 nm using a microplate reader (Epoch 2; BioTek Instruments). Each experiment was performed in triplicate, and the percentage of cell viability was determined as the ratio of the absorbance values of the experimental samples to the control solvent (DMSO) on the same plate.

### JEV and DENV2 infection and compound treatment

BHK-21 cells were seeded in a 24-well cell culture plate at a density of (0.25 × 10^6^ cells/well) with regular growth medium containing DMEM + 10% FBS 1 day prior to infection in triplicate. Once the cells reached 85%–90% confluence, they were infected with DENV2 and JEV virus inoculum comprising DMEM + 2% FBS and 10 µM of the compound at an MOI of 0.1. Cells were incubated for 1.5 h at 37 °C with the virus-compound inoculum, with gentle rocking every 10 min to ensure even coverage and prevent the cellular monolayer from drying. Finally, the inoculum was replenished with fresh culture media comprising DMEM + 2% FBS and the compound, and then kept in an incubator in a humidified chamber at 37 °C until harvest (18 h). The cell culture supernatant was aspirated and washed with PBS, and the cellular monolayer was used for viral RNA isolation.

### RT-PCR and quantitative PCR

Total cellular RNA was extracted from BHK-21 cells using TRIzol reagent, according to the manufacturer’s instructions (Ambion Life Technologies, USA). RNA quantity and integrity were determined prior to use on a NanoDrop spectrophotometer (Thermo Fisher Scientific). Then, 1 µg of total RNA from each experimental sample was used to synthesize cDNA by reverse transcriptase (RT) by incubating the RNAs with Moloney murine leukemia virus (M-MuLV) RT, with specific RT primer, 5′-ACAGTCTTTCCTTCTGCTGCAGGTCT-3′ (*T*_m_ = 63 °C), and deoxynucleoside triphosphates (dNTPs) (New England Biolabs) at 42 °C for 50 min, followed by inactivation at 70 °C for 15 min.

To quantify the viral RNA copy number, quantitative RT-PCR (qRT-PCR) was performed in duplicate using iTaq Universal SYBR Green Supermix (Bio-Rad). The cDNAs used for qPCR analysis were prepared using Power SYBR Green PCR Master Mix (Bio-Rad, #172–5125) in a QuantStudio5 Applied Biosystems real-time PCR system. For qPCR amplification of the JEV amplicon size of 202 bp, the sequences of the forward and reverse primer pairs used were 5′-GAGAGAGGCGCGCCAGTGGAAGGCTCAGGCGTCCAAAAG-3′ (*T*_m_ = 65 °C) and 5′-ACAGTCTTTCCTTCTGCTGCAGGTCT-3′ (*T*_m_ = 63 °C) and for DENV amplicon of 163 bp, the sequences of the forward and reverse primer pair used were 5′-TAGCGGTTAGAGGAGACCCCTC-3′ (*T*_m_ = 61 °C) and 5′-GAGACAGCAGGATCTCTGGTCT-3′ (*T*_m_ = 59 °C). The viral amplicon levels were normalized to those of mouse/human glyceraldehyde 3-phosphate dehydrogenase (GAPDH) using the forward and reverse primers 5′-CATCACTGCCACCCAGAAGACTG-3′ and 5′-ATGCCAGTGAGCTTCCCGTTCAG-3′. The average threshold cycle (*Ct*) values were converted to viral copy numbers by comparison with a standard plot established using known amounts of JEV cDNA. ΔCt = (*Ct* target mean *Ct* of reference genes). Delta *Ct* for both control and compound-treated samples was determined and subsequently analyzed by determining the delta *Ct* to determine the mRNA expression of target genes in control vs treated samples.

### Time of drug addition assay

BHK-21 cells were seeded in a 12-well cell culture plate at a density of (0.5 × 10^6^ cells/well). Upon 85%–90% confluency, cells were infected with JEV virus at an MOI of 0.1. Cells were incubated for 1.5 h at 37 °C. Infected cells were treated with 10 µM of each compound at different time points: pre + during + post (−3), during + post (0) and post (3, 6, 9). Cells were harvested at 24 hpi, and the viral genomic RNA levels were quantified using qRT-PCR.

### Determination of viral titer by TCID_50_ assay

During DENV2 and JEV infection, the viral supernatant was collected and used in the TCID_50_ assay to determine the viral titer. Then, 3 × 10^4^ BHK-21 cells per well were seeded in a 96-well cell culture microtiter plate the day before titration. The virus stock was diluted with infection media (DMEM + 2% FBS) to attain virus dilutions of 1 × 10^−2^ to 6.4 × 10^−7^ in 12 parallels, with the last column of the 96-well plate serving as virus-free cell control. On the day of the assay, when the cell had reached 90%–95% confluency, the growth medium was aspirated using a multichannel pipette, and 100 µL of virus inoculum of different dilutions was transferred to each well, keeping the last well only in infection media without virus. The cells were then incubated at 37 °C in a humidified 5% CO_2_ chamber. The 96-well plates were examined under a light microscope 48 hpi for signs of CPE. The infection medium was then removed, and the cells were rinsed with PBS to remove dead cells from the well surface. Cells were fixed with 70% ethanol for 20 min and counterstained with 2% crystal violet, followed by scoring of each well as positive or negative for viral growth. TCID_50_ was calculated using the Spearman and Karber methods.

### Molecular modeling

The crystal structure of DENV3 protease (PDB ID: 3U1I) (42) was downloaded from the Protein Data Bank (PDB). Chain C (NS2B) and chain D (NS3) were extracted, three residues (V36I, T115L, and K157R) were mutated, and renumbered to mimic DENV2 NS3 protease. Further it was prepared using Dockprep utility in UCSF Chimera-1.18 (57, 58) and saved as protein.mol2. Protein preparation includes a series of steps, such as water molecule deletion, addition of hydrogens, assignment of atom types (AD4), Gasteiger charge addition, application of selected flips to residues, and analysis of all-atom contacts. ChemDraw-19.1 was used to draw the 3D-geometry optimization and energy minimization of the most potent molecules (**3aq** and **3au**). Energy-minimized structures were saved in ligand.pdb format and further prepared using Dockprep utility in UCSF Chimera-1.18 and saved as ligand.mol2. Autodock Vina (59, 60) utility in UCSF Chimera-1.18 was used to perform docking. Grid center (35.96, –41.97, 46.10) was specified using the cocrystallized ligand (OAR) with box size of 20 × 20 × 20, energy range of 3, exhaustiveness of 8, and number of modes 10. Docked conformers were then analyzed using View dock utility in UCSF Chimera-1.18. Complex files were saved as complex.pdb for generating 2D-interaction plots using Ligplot+ (53).

### Bioinformatics analysis

Amino acid sequences of Dengue and Flavivirus whole genomes were retrieved from the NCBI virus database (https://www.ncbi.nlm.nih.gov/labs/virus/vssi/#/). 4,970 complete sequences of dengue virus and 8,821 complete sequences of different flavivirus (DENV, JEV, WNV, YFV, and ZIKV) were selected for the analysis. Multiple-sequence alignment was performed in the MAFFT version 7 server (https://mafft.cbrc.jp/alignment/server/) and further analyzed in Jalview 2.11.4.1 software. Logo plots were created using the WebLogo server (https://weblogo.berkeley.edu/logo.cgi) with aligned FASTA files of the NS3 catalytic core region from various flaviviruses, including DENV, JEV, WNV, YFV, and ZIKV.
